# Shock-Induced Damage Mechanism of Perineuronal Nets

**DOI:** 10.3390/biom12010010

**Published:** 2021-12-22

**Authors:** Khandakar Abu Hasan Al Mahmud, Fuad Hasan, Md Ishak Khan, Ashfaq Adnan

**Affiliations:** Department of Mechanical and Aerospace Engineering, The University of Texas at Arlington, Arlington, TX 76019, USA; mahmud.khandakarabuhas@mavs.uta.edu (K.A.H.A.M.); fuad.hasan@mavs.uta.edu (F.H.); mdishak.khan@mavs.uta.edu (M.I.K.)

**Keywords:** molecular dynamics, perineuronal net, shock loading, traumatic brain injury, cavitation

## Abstract

The perineuronal net (PNN) region of the brain’s extracellular matrix (ECM) surrounds the neural networks within the brain tissue. The PNN is a protective net-like structure regulating neuronal activity such as neurotransmission, charge balance, and action potential generation. Shock-induced damage of this essential component may lead to neuronal cell death and neurodegenerations. The shock generated during a vehicle accident, fall, or improvised device explosion may produce sufficient energy to damage the structure of the PNN. The goal is to investigate the mechanics of the PNN in reaction to shock loading and to understand the mechanical properties of different PNN components such as glycan, GAG, and protein. In this study, we evaluated the mechanical strength of PNN molecules and the interfacial strength between the PNN components. Afterward, we assessed the PNN molecules’ damage efficiency under various conditions such as shock speed, preexisting bubble, and boundary conditions. The secondary structure altercation of the protein molecules of the PNN was analyzed to evaluate damage intensity under varying shock speeds. At a higher shock speed, damage intensity is more elevated, and hyaluronan (glycan molecule) is most likely to break at the rigid junction. The primary structure of the protein molecules is least likely to fail. Instead, the molecules’ secondary bonds will be altered. Our study suggests that the number of hydrogen bonds during the shock wave propagation is reduced, which leads to the change in protein conformations and damage within the PNN structure. As such, we found a direct connection between shock wave intensity and PNN damage.

## 1. Introduction

Concussion, subconcussions, and exposure to an explosive blast’s shock waves can cause mild traumatic brain injury (mTBI) [1]. A typical blast-induced shock wave profile exhibits a sudden increase in pressure, often called overpressure, followed by a low magnitude with a longer duration negative pressure tail [2]. For example, a typical blast can generate an initial overpressure of over 27 GPa [3]. As a result, the shock wave from the blast can travel at a speed several times higher than the sound speed of the medium; for example, in an underwater explosion the shock wave can travel several thousand meters per second [4,5]. The long-range negative pressure tail causes damage to the extracellular matrix (ECM) and neuronal cells by forming a micro cavitation [2,6] and causing mechanical fracture of different biomolecules [6,7,8,9]. In addition, the overpressure generates a compressive load, which may cause shear fracture of biomolecules.

The perineuronal net (PNN) is a critical ECM component. It forms a safety net surrounding the neuronal cell body (soma) to give neuroprotection. It is an interconnected net-like structure consisting of three significant biomolecules: lectican, tenascin-R (TR), and hyaluronan (HA) [10]. The lectican family of the chondroitin sulfate proteoglycans (CSPGs) is the most prominent PNN of the central nervous system (CNS). Lectican consists of a core protein (CP) with covalently connected negatively charged glycosaminoglycan (GAG) side chains [11]. TRs are globular proteins that bind to the C-terminal domain of CP. HA is an unsulfated GAG synthesized by the hyaluronan synthase (HAS) enzyme [12]. This membrane-bound enzyme emanates through the plasma membrane into the extracellular space [13]. In addition, HA binds to the N terminal of other ECM protein molecules such as the CP and various link proteins (LPs) [14]. The PNN has many functional roles apart from neuroprotection. For example, it helps regulate synaptic plasticity and protects neuron cells from oxidative stress. Moreover, the proteoglycan molecules’ net negative charge helps control the diffusion of sodium, potassium, and calcium ions. As a result, a neuron with a PNN is fast-spiking with a narrower action potential than neurons without a PNN [15]. Therefore, the absence of a PNN in the neuron may cause a severe problem. Sometimes aberrations in its molecular structure also affect the functionality of the neuron. For instance, researchers have found that a reduction in normal PNN densities in the brain areas often affects cognitive functions in subjects with Alzheimer’s disease (AD) [16]. PNN loss in AD may contribute to altered excitatory/inhibitory balance, synaptic loss, and increased susceptibility to oxidative stress [11]. Some studies have also suggested that TBI is associated with an earlier onset of AD [17]. Thus, it is undeniable that structural alteration or loss of the PNN in the brain plays a critical role in neurodegeneration and cognitive functions.

The morphological degradation of the PNN from shock waves causes several diseases and alters the action potential by damaging the synapse. Propagation of the shock waves through the brain tissue damages the primary or secondary bonded structure of several PNN molecules. PNN molecules include proteoglycan, tenascin-R, link protein, and hyaluronan. Among them, HA, which is the backbone of the PNN, is more prone to damage [18]. Several studies have discussed the specific functions of HA in the ECM of brain cells. HA is a potential biomarker of injured brain tissues. Studies have found that cerebrospinal fluid (CSF) HA levels increase in patients with TBI [19]. Upon brain injury CD44 (hyaluronan receptor) appears around the lesion. HA promotes the migration of health-monitoring glial cells such as astrocytes. It also accumulates at the lesion and activates microglia (another type of glial cell) to initiate phagocytosis [19]. In contrast, proteoglycan and tenascin-R promote the structural integrity of the PNN network [20,21]. Therefore, investigating the behavior of the PNN under shock loading is essential.

The shock wave can break the PNN in the presence of nanobubbles. Studies have shown that shock velocity and cavitation bubble size affect the fracture potential of the ECM [18,22]. The higher shock speed and bigger bubble size are more damaging [18,22]. A cavitation bubble is defined as a bubble in liquid formed by pressures below the vapor pressure of the liquid. Several studies have been conducted on the shock wave effect on the transport phenomena and the deformation strain of the lipid bilayer [23,24,25]. However, the deformation mechanism of the PNN component due to the shock wave has not been studied yet. Shock can damage the PNN molecules by compressive shear force and negative tensile pressure that causes cavitation in the system. The timescale for cavitation formation due to shock propagation is not within the scope of an MD simulation study. There is always a possibility of preexisting bubbles in the ECM. Therefore, in this study, the collapsing preexisting bubble effect on the damage efficiency was emphasized. Depending upon the collapsing criteria, damage to the PNN may differ. The bubble may collapse symmetrically or asymmetrically. To determine how a shock interacts with a cavitation bubble and the surrounding liquid, the timescale for the shock wave passing-time and bubble collapsing time should be evaluated. If the collapsing time and shock wave passing-time ratio is less than one, the bubble will collapse asymmetrically. The equation of shock wave passing-time and collapsing time can be found elsewhere [18,22]. The preexisting bubble causes damage by forming a water jet [18,22]. While interacting with the shock front, the bubbles impinge on the PNN network molecule and cause damage to the network. The experimentally stable bubble is microscopic, whereas a nanosize bubble is often used in MD simulation studies [18,22]. Maximum overpressure generated from an explosive blast is in the 100 KPa to 50 MPa range [26,27]. Often a nanosize bubble is used in an MD simulation study, which is 10 to 100 times smaller than the experimentally observable bubble. The kinetic energy of the water-hammer jet is related to the bubble size and the post-shock pressure of an ideal planar shock wave through:

(1) $$E_{k}\infty D^{3}P_{p}$$

where, $E_{k}$, D, and $P_{p}$ are denoted as the total kinetic energy of the water hammer, diameter of the bubble, and post-shock pressure, respectively.

The brain comprises highly heterogeneous materials, consisting of different biomolecules, such as proteins, carbohydrates, GAGs, etc. Therefore, the effect of propagating shock has additional damage efficiency on different types of molecules. Depending on the size of the molecule and molecular weight, the mean square displacement (MSD) of various molecules varies at the same shock speed [28,29,30]. Moreover, some molecular chains are incredibly long compared to others. Therefore, the acceleration level at the nanoscale in each part of the long chain will be different. Because of the differential acceleration in each part of the long chain, differential pressure exists, and thus the potential to fracture is high for long-chain molecules. A recent study found that cavitation collapse creates a water-hammer jet that potentially causes damage to the ion channel protein. Even at a lower shock speed, separation of the protein from the lipid bilayer has been observed [29]. The mean square displacement (MSD) of the ion channel protein is higher than the lipid bilayer. Interconnection between them plays an important role in the displacement from shock wave propagation. Similarly, the interfacial strength and morphology of the individual PNN molecules play a critical role in damaging individual molecules. For example, proteins are more rigid molecules consisting of primary, secondary, and tertiary structures [9,31,32]. The shock wave can hardly damage the primary structure of the protein. It mostly causes damage to the secondary and tertiary structures. Because of the folding capability of the proteins, they can self-heal by folding to their secondary and tertiary structure [33,34]. Other molecules of the PNN, such as hyaluronan, do not have secondary and tertiary structures. Thus, they are more prone to damage from shock waves and sustain damage without healing.

In this study, different junctions of the PNN, which are noncovalently bonded, are studied to assess their relative strength by steered molecular dynamics simulation (SMD) techniques. There are three interfaces among various components of the PNN, such as

1. The link protein (LP) and core protein (CP) of proteoglycan (PG),
2. Tenascin-R (TR) and CP,
3. The LP and hyaluronan (HA),

The relative strength of the junctions was evaluated to find the weakest link among the molecular interfaces of the PNN conjugate. Afterward, the damage efficiency of the shock wave on the PNN model is studied.

## 2. Methodology

### 2.1. Modeling of the PNN Structure

**Figure 1:**
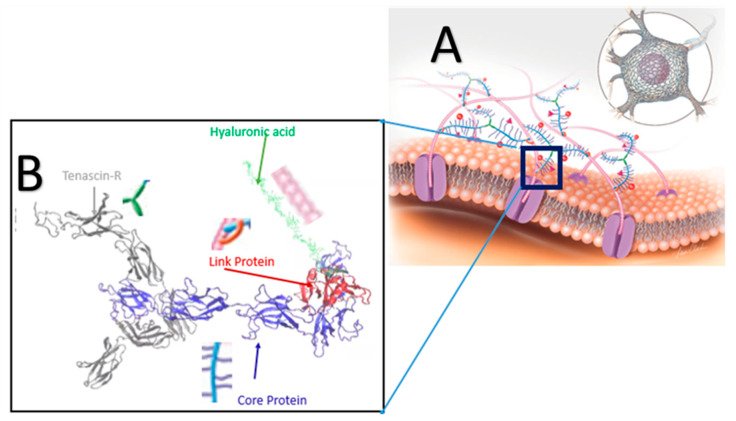
(**A**) Schematic illustration of the PNN structure. The overall macromolecular structure of the PNN is obtained by the specific arrangement and binding of the components [16]. A major component of the PNN is chondroitin sulfate proteoglycans (CSPGs) that include a core protein (CP) (blue) and several sugar chains (purple). Structurally, the CPs are bound to hyaluronic acid (HA) (pink). A set of link proteins (LPs) (orange) are also present in the PNN to stabilize the interaction between HA and CPs. Sema3A (pink pyramids) and Otx2 (red balls) are linked with the sugar chains of the CSPGs. Tenascin-Rs (green) are proteins in the PNN that are cross-linked with the CSPGs. (**B**) Docked PNN model structure.

The perineuronal-net (PNN) is a protective ECM component surrounding the neuronal cell (Figure 1A). The basic building blocks of the PNN include hyaluronic acid (HA), proteoglycans (PGs), tenascin-R (TR), and link protein (LP) (Figure 1B). Proteoglycan consists of core protein (CP) and glycosaminoglycan (GAG) chains connected to the core protein via a glycosidic covalent bond. GAG chains of the CP are negatively charged. Therefore, these GAG side chains help balance the charge distribution of neuronal cells. These small GAG chains contribute less to the mechanical stiffness of the PNN; thus, in this study, GAG chains were omitted. The PNN structure consisted of HA, TR, CP, and LP in this study. The TR and CP were connected by non-covalent bonding, HA and CP by non-covalent or glycosidic covalent bonds mediated by an LP. Docking protocol was used to model the most energetically favorable protein complex. The ClusPro online server was used [28] to perform molecular docking for protein–protein docking. ClusPro introduced PIPER, an FFT-based docking program. It uses a pairwise interaction potential as part of its scoring function *E*, where *E* is

*E* = *E*_attr_ + *w*_1_ *E*_rep_ + *w*_2_ *E*_elec_ + *w*_3_ *E*_pair_. (2)

*E*_attr_ and *E*_rep_ denote the attractive and repulsive contributions to the van der Waals interaction energy *E*_vdw_. $E_{\mathrm{elec}}$ is an electrostatic energy term. *E*_pair_ represents the desolvation contributions. The coefficients *w*_1_, *w*_2_, and *w*_3_ specify the related terms’ weights and were optimally selected for different docking problems [35].

At first, the LP and CP were docked, then the LP–CP protein complex was further anchored with TR to get the final protein complex (LP, CP, and TR) of the PNN. Finally, a hyaluronic acid chain was attached to an LP by a glycosidic covalent bond using the CHARMM-GUI Glycan Reader and Modeler module [36]. The HA chain consisted of 15 repeated dimers of β-N-Acetylglucosamine (GlcNAc) and β-D-glucuronic acid (GlcA) linked via alternating β-(1→4) and β-(1→3) glycosidic bonds [37]. The PNN unit is shown in Figure 1B; it represents the inset portion of Figure 1A.

### 2.2. Interfacial Strength of PNN Components

**Figure 2:**
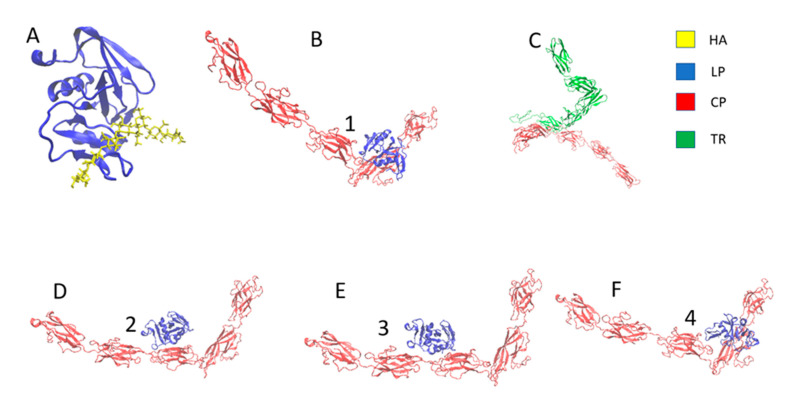
Docked structure (**A**) HA–LP complex, (**B**) LP–CP complex, and (**C**) CP–TR complex. (**B**,**D**–**F**) Top 4 energy-minimized structures of the LP–CP complex (proteins are represented in new cartoon representation and hyaluronan is represented in bonded representation and 1, 2, 3 and 4 denotes first four energy minimized structure of LP-CP complex).

The ClusPro server was used for the protein–protein docking (Figure 2B,C), and the LP–HA (Figure 2A) complex was taken from the original PDB structure of the HA binding domain of murine CD44 from the RCSB protein data bank (PDB ID: 2JCQ) [38]. ClusPro generated 100 energy-minimized structures; only the most energy-minimized configuration was taken for this study. The minimum energy configuration is shown in Figure 2B,C. Figure 2B,D–F shows the CP–LP complex’s top 4 energy-minimized structures.

Three complex structures were tested for the relative interfacial strength calculation using the steered molecular dynamic (SMD) approach in the GROMACS 5.0 simulation platform [39,40,41]. The LP molecule was fixed of the LP–HA protein–ligand complex and pulled the HA at a constant speed, while for the LP–CP and CP–TR protein–protein complexes, the LP and TR were pulled at a steady rate. In addition, the molecules were pulled from their center of mass.

### 2.3. Mechanical Property Evaluation of PNN Components

**Figure 3:**
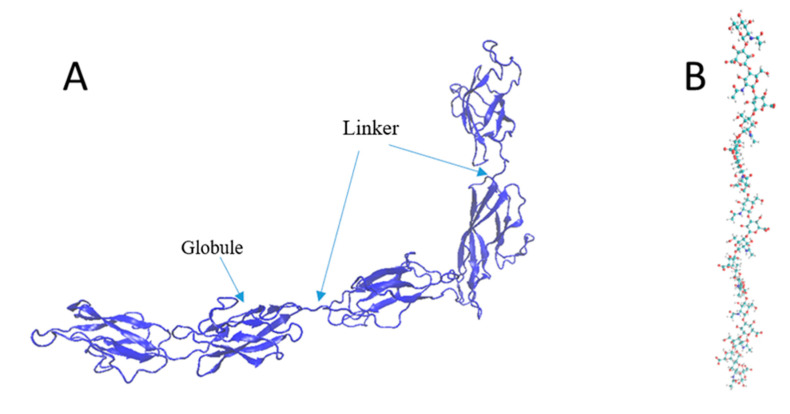
Structure of (**A**) the CP (new cartoon representation) and (**B**) HA (all-atom representation).

The mechanical strength of the components is vital to evaluating the underlying mechanics of the PNN under shock waves. Since one of the significant functions of the PNN is to provide neuronal protection from mechanical damage, the interfacial strength of the protein complex and the individual strengths of the components needed to be evaluated. This study assessed the CP and HA mechanical properties using the CHARMM36 and ReaxFF force fields, respectively. Because of the CP and TR’s structural conformational similarity, we assumed that the CP and TR deformation profiles would be similar. Because of the globular tertiary structure, the primary covalent bond break was very unlikely for the protein molecules. Instead, the applied force will cause secondary and tertiary structural failure. Two tertiary globules of the CP were connected by a chain (Figure 3A). The whole structure contained a single chain, and the secondary structure contained an alpha-helix and beta-sheet. In comparison, the HA single chain had 10 dimers (Figure 3).

The SMD approach evaluated the mechanical strength of the CP and HA. Few atoms at the end of the molecules were pulled at a constant velocity of 1000 ${\mathrm{ms}}^{-1}$. While the other end was fixed, the temperature was maintained constant at 310 K in all the simulations. In the beginning, the system was energy-minimized using the shaking algorithm. After that, the energy-minimized structure was equilibrated at the NPT ensemble, where temperature and pressure remained constant. Afterward, pulling simulation was conducted in the NVT ensemble. In NVT, volume and temperature remained constant.

### 2.4. Shock Simulation

**Figure 4:**
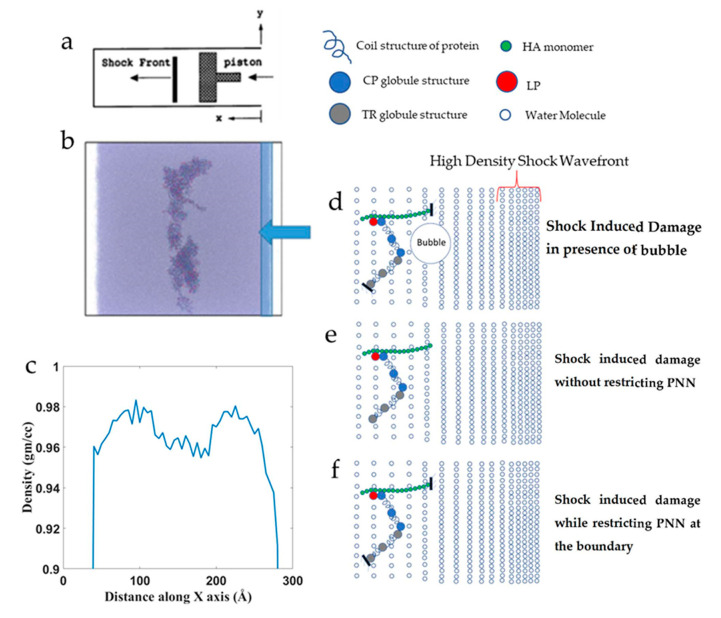
The simulation box for shock propagation. (**a**) Schematic illustration of the shock simulation setup. (**b**) Snapshot of the shock simulation box by the OVITO visualization tool. (**c**) Density profile along the shock direction. (**d**–**f**) Schematic illustration of the simulation protocol (**d**) while restricting the PNN in the presence of bubble, (**e**) without restricting the PNN, and (**f**) with restriction.

The PNN structure in Figure 1B was used to conduct the shock simulation. The PNN model was solvated with TIP3P water and ions (0.1 M NaCl) using the CHARMM-GUI Glycan Reader and Modeler [36] module. The box size was 26.2 × 26.2 × 26.2 ${\mathrm{nm}}^{3}$ filled with water and PNN molecules. The X-direction was the shock wave propagation direction. The shock wave was formed from the positive direction and propagated to the negative X-direction. Both ends of the normal box to the X-directions were opened up to create a vacuum space. It was possible to restrict the shock flow to the opposite end because the periodic boundary condition was applied along the shock direction. There are different ways of generating shock waves, one of the most common methods is the “moving piston” [42] and the “reflecting boundary” is another popular method that is widely used [18,22,43]. The piston-driven shock has several advantages over the reflecting boundary method. The initial number of particles in a cell remains constant throughout the simulation until the shock wave nears. Therefore, density remains constant in the upstream region. Secondly, the piston-driven shock wave simulation closely resembles the corresponding physical experiment. Even though assumptions are made on the nature of the particles’ interactions and between the particles and computational boundaries, the model can simulate otherwise difficult experiments. To initiate the shock, several layers of water molecules from the right end of the x-axis were made rigid and pushed at a constant velocity for a certain distance, and then the piston motion was halted (Figure 4b). The pistons were moved at 1, 2.5, and 4 km/s velocity for 30 Å distance. Density distribution of the simulation box showed that the region where the PNN network molecules were present showed a 2% reduced (0.96 gm/cc) density compared to water density (0.98 gm/cc) at 310 K (Figure 4c). We conducted the shock effect on the PNN network under three different conditions to understand the shock wave effect on a network structure. Figure 4d shows the shock propagated in the presence of preexisting bubbles, Figure 4e shows the shock effect when the network was not restricted at any point. Finally, Figure 4f shows the schematic of shock propagation when the PNN was restricted at several points. All visualizations of the simulations were done in VMD [44] and OVITO [45]. The number of hydrogen bonds was calculated using the VMD HBonds Plugin [44].

Pressure is an important parameter that we used to make an educated guess at a molecule’s failure during shock wave propagation. Therefore, it is essential to discuss the pressure calculation protocol. During simulation, each atom was placed in a Voronoi tessellation cell. Cell volume was the atomic volume. Details of the Voronoi tessellation cell algorithm are stated elsewhere [46,47]. Per-atomic virial stress was calculated from the following formula:

(3) $$S_{ab}=-mv_{a}v_{b}-W_{ab},$$

(4) $$W_{ab}=\frac{1}{2}\sum_{n=1}^{N_{p}}\left(r_{1a}F_{1b}+r_{2a}F_{2b}\right)+\frac{1}{2}\sum_{n=1}^{N_{b}}\left(r_{1a}F_{1b}+r_{2a}F_{2b}\right)+\frac{1}{3}\sum_{n=1}^{N_{a}}\left(r_{1a}F_{1b}+r_{2a}F_{2b}+r_{3a}F_{3b}\right)\;+\frac{1}{4}\sum_{n=1}^{N_{d}}\left(r_{1a}F_{1b}+r_{2a}F_{2b}+r_{3a}F_{3b}+r_{4a}F_{4b}\right)+\frac{1}{4}\sum_{n=1}^{N_{i}}\left(r_{1a}F_{1b}+r_{2a}F_{2b}+r_{3a}F_{3b}+r_{4a}F_{4b}\right)\;+K_{space}\left(r_{ia},F_{ib}\right).$$

The first term of Equation (3) is a kinetic energy contribution for atom I, and $W_{ab}$ is the potential energy contribution to the per-atomic virial stress $S_{ab}$. The first term of Equation (4) is from the van der Waals energy contribution; the second, third, fourth, and fifth terms are from the bond, angle, dihedral, and improper contributions, respectively. The last term is the charged potential contribution to the virial stress. Details of the formulation are stated in the LAMMPS manual [48]. Equation (5) calculates the hydrostatic pressure P from virial stress, where N is the number of atoms in the group. V is the volume of the atoms calculated from the Voronoi tessellation algorithm. This methodology can be utilized to calculate the pressure on a group of atoms such as individual components of the PNN, pressure on HA, CP, TR, and LP.

(5) $$P=\frac{\sum_{n=1}^{N}S_{ab}}{\sum_{n=1}^{N}V_{a}}.$$

## 3. Results and Discussions

### 3.1. Mechanical Strength of the PNN Components

The mechanical strength of the ECM components, such as the core protein (CP) and hyaluronic acid (HA), was measured by SMD simulation. The CP is a very long protein coil chain that forms a secondary structure known as alpha-helix and beta-sheet. These secondary structures are strong and bonded by interchain hydrogen, electrostatic, and van der Waals interactions. The secondary system breaks during the pulling simulation, which is primarily a non-covalent electrostatic bond. Covalent bond breakage of the protein structure is very rare. Therefore, it is wise to use a non-reactive CHARMM36 force field for the CP. The non-reactive CHARMM36 force field is advantageous over the reactive because it is widely used for biomolecules, and simulation time is faster than the ReaxFF reactive force field.

**Figure 5:**
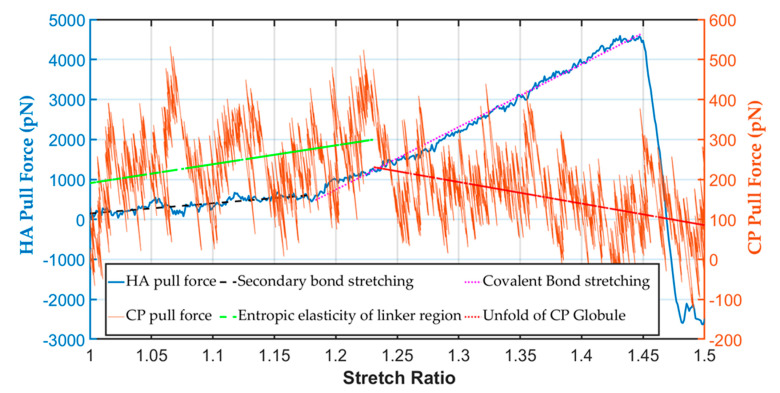
Mechanical strength of the PNN CP and HA components at 1 ${\mathrm{kms}}^{-1}$ pulling speed.

In, the CP was stretched to 50% of its initial length, and the maximum pulling force was only 400 pN. The maximum pull force for protein tertiary structure unfolding was in a range similar to that found experimentally using the AFM technique (500 pN) [49]. It was observed that the unfolding force varied in a sawtooth pattern with respect to the stretch ratio. Upon stretching, the force gradually increased owing to the entropic elasticity of the unstructured linker region between two globules (Figure 5). After that, as the tensile force rose, one of the globular domains could not resist the force and started to unfold. In our study, we saw such a pattern during the stretching of the protein. We found in this simulation that the linker regions elongated at 0 to 25% stretch from the unfolding observation. After that, an unfolding of the weakest globule started (at 25 to 50% stretch). The simulation data fluctuated significantly for the pull force of the CP; therefore, to find the trend of different regions, we fitted the data by linear curve fit. The maximum stretching force of the linker region from the fitted result was 300 pN, also within the experimental value.

In contrast, the maximum pulling force for hyaluronic acid at 40% stretch was around 4500 pN, more than 10 times the maximum CP stretched force. The HA covalent bond broke at above ~45% strain. From the force-displacement curve of HA (Figure 5), it was found that at the toe region, the secondary bond stretched, and after that, around 20% strain covalent bond stretching started and finally failed at 45% strain. Thus, the stiffness constant at the toe region was ~20 pN/Å. In contrast, at the covalent bond stretching region or elastic region, ~160 pN/Å, the stiffness constant was eight times higher at the covalent bond stretching part than the toe region. When the stable helical HA configuration was pulled out, it started straightening by breaking the non-covalent bonds in the toe region, and covalent bond stretching started. The covalent glycosidic bonds were the weakest junctions between the disaccharides of HA [37]. Thus, on shock loading or pull force, the glycosidic bonds broke. Unlike the protein molecules, such as the core protein and tenascin-R of the PNN, the failure strain of polysaccharides was low (Figure 5).

### 3.2. Interfacial Strength

**Figure 6:**
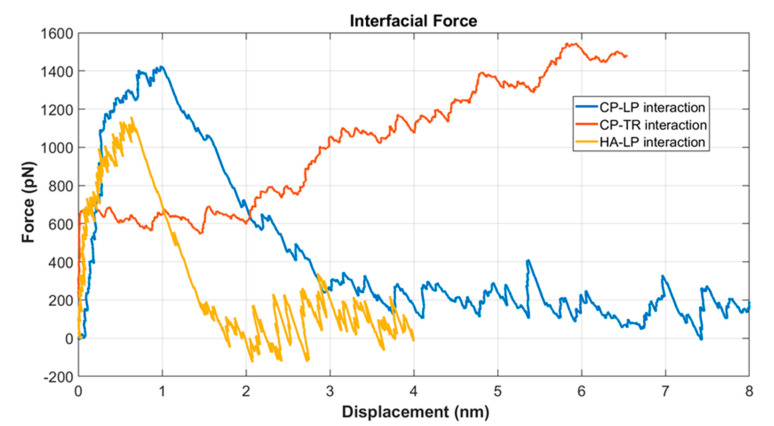
Interfacial strength of the PNN components at 1 ${\mathrm{kms}}^{-1}$ pulling speed.

In the PNN network, three different interfaces existed: CP–LP, LP–HA, and CP–TR. Interfacial strength played a significant role in the mechanics of the PNN under shock wave. LP, HA, and TR from the three interfaces were pulled, whereas the other molecules (CP, LP, and CP) of the pairs were kept fixed at their initial positions. The CP > TR > LP > HA mass for the PNN model, although in reality, the molecular mass of HA was maximum because of its very long chain. Figure 6 shows the relative interfacial strength of the three different interfaces. It was found that HA–LP had the lowest strength and CP–TR the highest. The CP–TR bonds never failed during the simulation. Instead, the TR molecules unfolded. The interfacial strength for the pair of molecules considered was between 1100 and 1500 pN. This strength was well below the fracture strength of the covalent bond. The LP–HA interface failed at around 1100 pN force, whereas the LP–CP failed at 1400 pN force. The CP–TR did not fail during the simulation period.

In the literature, the dissociation of proteins is often shown in terms of the potential of mean force (PMF) and free energy change (ΔG) of the system [50]. However, since the shock wave was a mechanical energy dissipation in liquid, we wanted to quantify the high strain rate dissociation force between the protein/protein and protein/HA interface. One molecule was pulled along a reaction coordinate in the SMD, whereas the other was restricted at the initial equilibrated position [51]. Interestingly, the three pairs of molecules pulled instantly showed a steep increase in force. After that, the slope decreased, and finally, separation took place, and force decreased. We considered the biomolecules in water to be a composite structure. Water molecules adhered with the protein and HA by non-covalent bondings. When pulled, the bondings between the water and biomolecules (protein and HA) broke. Therefore, the force’s instant increase was considered a pull-out interfacial shear force [52]. Further separation of the interface caused the interfacial bondings between the biomolecules to break. Salt bridges between the proteins were responsible for the strong adhesion force. The bonds included the hydrogen bonds and other non-covalent bonds formed between oppositely charged residues [53].

### 3.3. Shock Simulation

**Figure 7:**
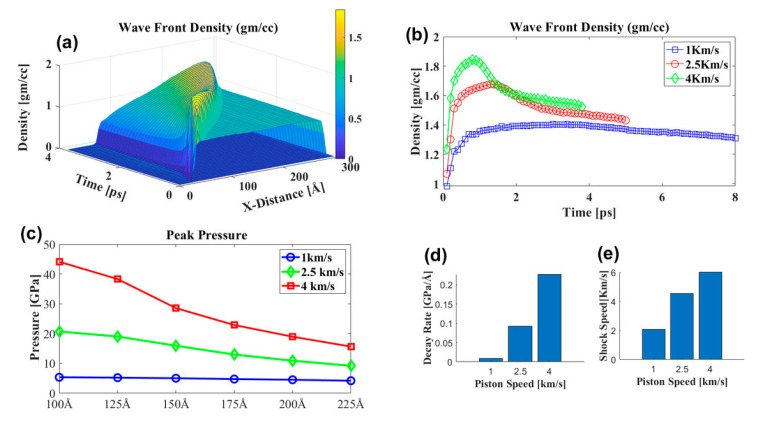
(**a**) Shock wavefront density at different piston speeds. The inset plot shows the piston speed’s corresponding shock velocity. (**a**,**b**) Density distribution during the shock propagation at different locations and times for 4 km/s piston speed, the color bar represents density variation along the shock direction. (**b**) Shock wavefront density at different piston speeds. (**c**) Peak pressure at different locations along the x-axis during the shock propagation at different shock speeds. (**d**) shows the maximum pressure decay rate at different shock speeds represented in (**c**). (**e**) shows the piston speed’s corresponding shock velocity.

In this work, a piston was used to initiate the shock. The piston was moved up to 30 Å from the positive x-axis at 1, 2.5, and 4 km/s speed to create different shock speeds. Figure 7e shows the corresponding shock velocity at different piston speeds.

#### 3.3.1. Effect of Shock Speed

The shock wavefront was highly densified, which is called the overpressure region. After the overpressure region, there was a sharp decrease in density; the density profile over time along the shock propagation direction is shown in Figure 7b. The simulation box was divided into 61 bins along the shock propagation direction, and each bin was 5 Å in size. The average properties, such as velocity, density, and pressure, were calculated for each bin’s particles. As the shock propagated, the maximum density decayed, and the decay rate at the higher piston speed was much higher (Figure 7d). The peak pressure at different bin locations in Figure 7c shows that maximum decay was observed at 4 km/s piston speed, and at 1 km/s, minimum pressure decay along the shock direction. At the simulation box’s middle sizes (125 to 150 Å), the decay rate was higher for 4 and 2.5 km/s piston speeds. However, no significant change in decay constant was observed at 1 km/s piston speed. The PNN molecules in the middle of the simulation box may have impeded the water molecules’ motion as the shock propagated. Thus, this region’s peak pressure dropping rate was highest compared to the other areas. However, the penetration of water molecules was not hindered by the presence of PNN molecules at 1 km/s. Therefore, no significant change in the peak pressure was observed in this region; this implied that water molecule penetration efficiency depends on the pressure impulse. At higher pressure, the impulse penetration rate was lower [24]. Shock impulse attenuation was experimentally observed at different concentrations and gel thicknesses of the methylcellulose (MC) hydrogels by Orel et al. [54]; as the gel concentration and thickness increased, shock impulse attenuation increased. The shock attenuation rate was lowest for water compared to the different concentrations of MC hydrogels, which suggested that internal microstructure plays an essential role in the shock propagation and damage mechanism of the PNN. The network hindered the shock propagation by absorbing the shock energy, which will cause damage to the network microstructure.

**Figure 8:**
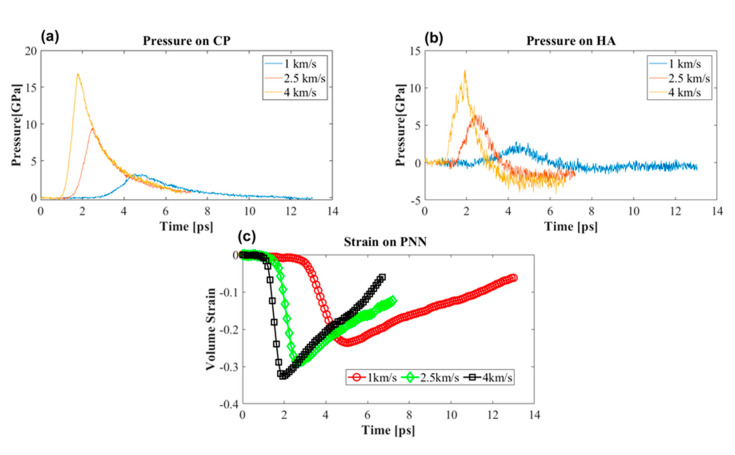
Pressure on the PNN components at different piston speeds. (**a**) Pressure on the core protein (CP). (**b**) Pressure on hyaluronan (HA). (**c**) Volumetric strain on the PNN at different shock speeds.

While the shock propagated, different molecules experienced different levels of pressure. Figure 8a,b shows the pressure profile in the CP and HA. These two molecules were considered because other molecules are proteins. They were representative of the structural and bonding conformation of the CP molecule. Therefore, it was reasonable to assume that the LP and TR would experience pressure similar to what the CP experienced. Peak overpressure depended on the shock speed; as the shock propagated the CP experienced 17, 9, and 3.5 GPa compressive stress, while HA experienced 12.5, 6, and 2 GPa at 4, 2.5, and 1 km/s piston speeds. With this compressive stress the PNN experienced a maximum of 35% compressive volumetric strain at 4 km/s (Figure 8c).

Interestingly, the CP did not experience any tensile stress, while HA experienced tensile peak pressures of around −5, −3, and −1 GPa. This study did not confirm whether or not HA would break. However, it was reasonable to approximate the cross-sectional area of HA around 50 to 100 ${\text{Å}}^{2}$; at this cross-sectional approximation, the failure stress of HA would be around 9 to 4.5 GPa. This value corresponded to the failure force of the HA in Figure 5. The approximate value of fracture stress suggested that 4 km/s piston speed corresponded to 6 km/s shock speed, and HA would most likely break. Because the average pressure of all atoms of the HA molecule was considered, there was a possibility that localized pressure would surpass the fracture stress at the rigid junction even at a lower shock speed.

#### 3.3.2. Effect of Cavitation Bubble

Research has been conducted on the effect of the bubble on the damage mechanics of biomolecules under shock loading [18,22,55]. When a shock wave passes through the cavitation bubble, the post-shock pressure compresses the anterior side of the bubble. The mass density of the bubble is much less than the surrounding liquid, which enables post-shock pressure to speed up bubble collapse, with the speed of the collapse even faster than the shock wave. The collapse of the bubble can be symmetric or asymmetric, depending on the ratio of collapsing time $t_{c}$ and shock passing time $t_{sp}$. If the ratio ($\frac{t_{c}}{t_{sp}}$) is higher than one, the bubble will collapse symmetrically, otherwise, an asymmetric collapse will be initiated. The ratio is related to the bulk modulus $\left(B_{L}\right)$ and peak pressure difference (Δp in Equation (6)). The bulk modulus of water is around 2.2 GPa, which means the cavitation bubble in water can only be asymmetrically collapsed by a shock wave with higher than 2.2 GPa post-shock pressure or near that scale [22].

(6) $$\frac{t_{c}}{t_{sp}}=\sqrt{\frac{B_{L}}{\Delta p}}.$$

**Figure 9:**
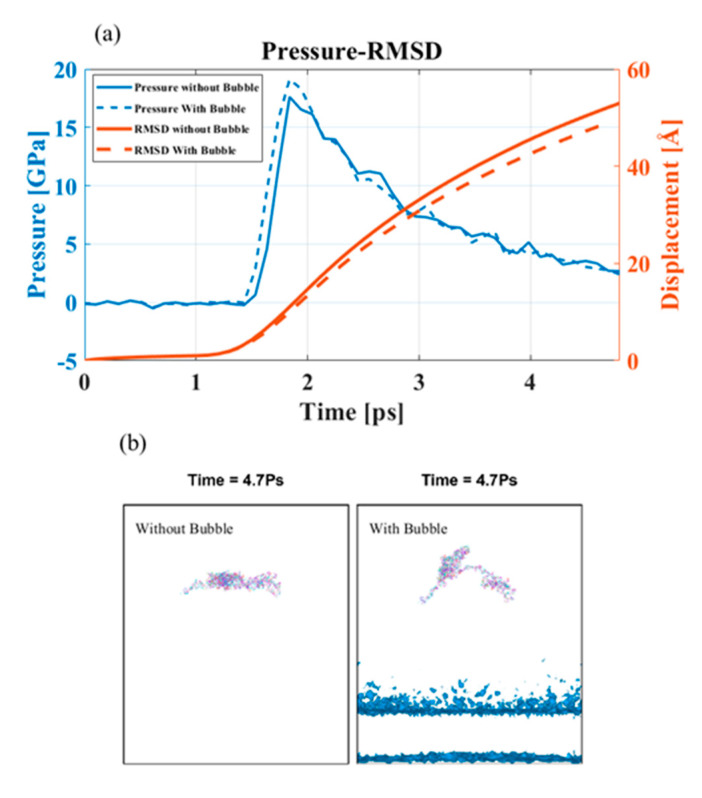
Bubble-induced shock propagation (**a**) Pressure on the bubble-projected area and RMSD value. (**b**) Bubble-projected area of the PNN at 4.7 ps in presence and absence of bubble.

In this case, the peak pressure was much higher than 2.2 GPa; it was reasonable to assume that the bubble would collapse asymmetrically. The bubble’s asymmetric collapse formed a water jet that reached farther away from the cavitation epicenter and caused more damage. We investigated the pressure profile of the PNN components in the presence of a bubble of 8 nm diameter and found an insignificant change in overpressure (Figure 9). However, the overpressure on the CP molecule bubble-projected zone was higher than that without the bubble model for 4 km/s piston speed (Figure 9a). However, it was interesting to note that although there was a differential overpressure at the projected area, the RMSD of the PNN was lower with a bubble at the same shock speed. A water jet formed during the bubble collapse mainly damaged the local region of the PNN. The portion of the CP molecule on the bubble-projected zone is shown in Figure 9b. The damage intensity can be very high, so the protein molecule’s primary structure was also susceptible to this damage. The model’s difference in deformation behavior with and without a bubble clearly showed that high water jet velocity can break any bond encountered (Figure 9).

#### 3.3.3. Effect of Boundary Conditions

**Figure 10:**
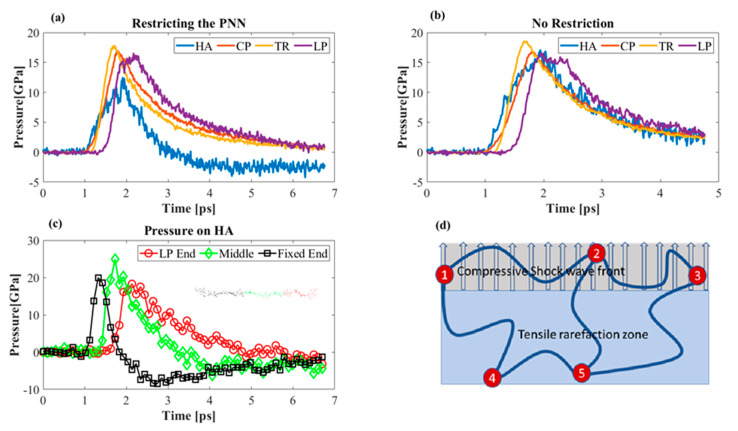
Pressure profiles of the PNN components. (**a**) Pressure on different PNN components without any restraint. (**b**) Pressure on various PNN components in the presence of boundary conditions. (**c**) Pressure at different locations of HA. (**d**) Schematic illustration of the PNN network structure.

Covalent and non-covalent bonds interlinked the PNN components (electrostatic and van der Waals bonding). There will be a difference in the acceleration profiles of the components. Therefore, it was reasonable to assume that one part of the molecule may experience 15 GPa while other parts are still at atmospheric pressure. The links are often considered rigid as compared to the molecule itself. We observed that only the HA molecule experienced negative tensile stress owing to fixing one end. Although two atoms at the two ends of TR were fixed, its average pressure did not change significantly (Figure 10a,b). HA mostly experienced tensile stress from its chain conformation. The globular structure of the proteins unfolded upon loading by breaking the non-covalent bonds. We found that the fixed end of HA experienced higher tensile pressure than the middle portion and the part connected to the LP (Figure 10c). HA was the backbone of the PNN. Therefore, it was essential to analyze its deformation behavior during shock propagation. We assumed the PNN to be an interconnected polymer network model similar to the schematic in Figure 10d. In an actual PNN, the CP and TR are responsible for the mechanical integrity of the network, and HA forms the backbone of the network. The previous study showed that a mere shock wave does not provide the necessary tensile force to damage the HA [18]. Instead, the presence of a bubble interacts with a shock wave, causing damage to the HA by the water jet. We argue that the actual ECM is a connected network structure (Figure 10d), bonded at the nodes (indicated as a red dot in Figure 10d). While some portion of the long HA chain was located in the compression zone during shock propagation, the other part was found in the tensile pressure zone of the model. The differential pressure and velocity profile of the atoms in each location caused a tensile pressure to the long-chain HA molecule. Nodes 1, 2, and 3 (Figure 10d) were located in the compression zone, whereas the Nodes 4 and 5 were located in the tensile zone. Shock propagated in the medium predominantly by the water molecules. If we applied the momentum balance equation, the net velocity of the water molecules would be much higher than that of the other biomolecules in the model [56]. We know that shock is a longitudinal pressure wave, while it propagates in heterogeneous media like ECM, the differential velocity of the water and biomolecules creates a tensile force to the long molecular chains. The shock wave passed through Nodes 1, 2, and 3 at 5 km/s wave speed, but Nodes 4 and 5 of the rarefaction zone remained at a lower velocity. This differential wave speed caused a tensile force to the connected molecule (the molecule between Nodes 2, 5; Nodes 1, 4; and Nodes 3, 5). We observed this phenomenon in our simulation. We restricted the HA molecules in one end to represent a bonded connection between the HA-producing enzyme hyaluronan synthases (HAS). While the shock wave propagated and hit the HA–LP junction, the restricted zone remained at a standstill, and the velocity difference caused a tensile strength to the molecule. The tensile pressure was sufficient to break the molecule (Figure 10c). Pressure at the LP–HA junction was compressive, whereas it was tensile near the fixed end of HA.

**Figure 11:**
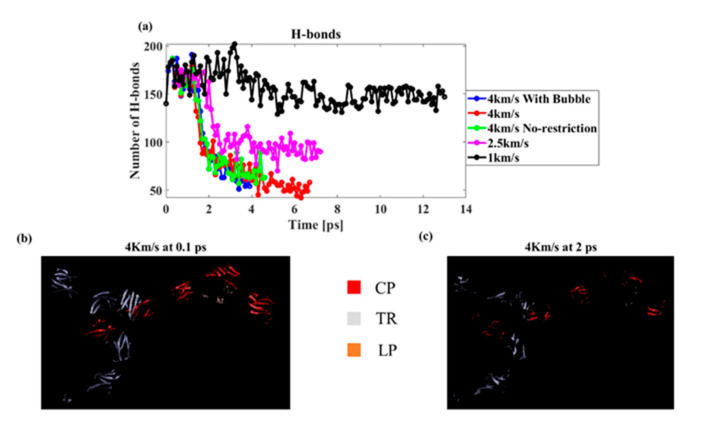
(**a**) Number of hydrogen bonds at different speeds and boundary conditions (cut-off distance 3 Å and angle 20°), (**b**) Beta-sheet at the initial undeformed state, and (**c**) beta-sheet in the deformed state due to the shock wave.

Finally, the number of hydrogen bonds was measured under different conditions (a). As the shock speed increased, the number of hydrogen bonds decreased. A hydrogen bond was found only in the protein molecules of the PNN, suggesting that the shock wave damaged the protein’s secondary structure. The lowest hydrogen bond was found for the model with the preexisting bubble, suggesting that the bubble jet caused maximum damage to the PNN. To further confirm the secondary structure damage, we visualized the beta-sheet of the PNN proteins using the VMD visualization tool. We found that the beta-sheet percentage was significantly reduced as the shock interacted with the protein structures (Figure 11b,c). Figure 11b,c shows the beta-sheet present at the initial undamaged PNN (0.1 ps) and during shock wave propagation, while the maximum hydrogen bond degraded (2 ps). It is clear from the image that the secondary structure was severely damaged from the shock propagation.

## 4. Conclusions

The PNN network protects the neurons from physical damage, reduces oxidative stress, and conserves charge balance to facilitate neurotransmission. Therefore, the probability of damage to the PNN under shock loading needed to be evaluated. From the shock loading simulation, it was concluded that:

The protein structure is less prone to failure from shock loading, while hyaluronan is the molecule most vulnerable to breakage during the shock loading.

The damage efficiency is strongly dependent on the shock speed, presence of a bubble, and boundary condition. A bubble in the system initiates asymmetric collapse during shock propagation and produces water jets that damage the molecules present in the projected domain.

Although the protein components’ pressure is still compressive, the significant reduction in the number of hydrogen bonds of the proteins clarifies that their secondary structure, such as the beta-sheet, alters significantly at higher shock speed.
